# Author Correction: Sublimed C_60_ for efficient and repeatable perovskite-based solar cells

**DOI:** 10.1038/s41467-025-55988-7

**Published:** 2025-01-20

**Authors:** Ahmed A. Said, Erkan Aydin, Esma Ugur, Zhaojian Xu, Caner Deger, Badri Vishal, Aleš Vlk, Pia Dally, Bumin K. Yildirim, Randi Azmi, Jiang Liu, Edward A. Jackson, Holly M. Johnson, Manting Gui, Henning Richter, Anil R. Pininti, Helen Bristow, Maxime Babics, Arsalan Razzaq, Suman Mandal, Thomas G. Allen, Thomas D. Anthopoulos, Martin Ledinský, Ilhan Yavuz, Barry P. Rand, Stefaan De Wolf

**Affiliations:** 1 King Abdullah University of Science and Technology (KAUST), KAUST Solar Center (KSC), Physical Science and Engineering Division (PSE), Thuwal, 23955-6900 Kingdom of Saudi Arabia; 2 Department of Electrical and Computer Engineering, Princeton University, Princeton, NJ 08544 USA; 3 Department of Physics, Marmara University, Istanbul, Türkiye; 4 Laboratory of Nanostructures and Nanomaterials, Institute of Physics, Academy of Sciences of the Czech Republic, v. v. i., Cukrovarnická 10, Prague, 162 00 Czech Republic; 5 Nano-C, Inc., 33 Southwest Park, Westwood, MA 02090 USA

**Keywords:** Solar cells, Electronic materials

Correction to: *Nature Communications* 10.1038/s41467-024-44974-0, published online 24 January 2024

The original version of this Article omitted the authors Suman Mandal and Thomas D. Anthopoulos from the King Abdullah University of Science and Technology (KAUST), KAUST Solar Center (KSC), Physical Science and Engineering Division (PSE), Thuwal, 23955-6900, Kingdom of Saudi Arabia.

Consequently, the following was originally omitted from the Author Contributions: ‘S.M. and T.D.A. fabricated the OFET devices and extracted the data. A.P. explained the OFET and mobility measurements.’

Additionally, the following was originally omitted from the Acknowledgements: ‘T.D.A. acknowledges the baseline funds provided by the King Abdullah University of Science and Technology.’

This has been corrected in both the PDF and HTML versions of the Article.

