# Supplementary material for: Sublimed C60 for efficient and repeatable perovskite-based solar cells
Source: Nat Commun. 2024 Jan 24;15:708. doi: 10.1038/s41467-024-44974-0 (PMC10808237; doi:10.1038/s41467-024-44974-0)
Supplement: Supplementary file 1 — Supplementary Information [file 41467_2024_44974_MOESM1_ESM.pdf]

Supplementary Information for

Sublimed C_{60} for efficient and repeatable perovskite-based solar cells

Ahmed A. Said[1,⊥,*], Erkan Aydin[1,⊥,*], Esma Ugur[1], Zhaojian Xu[2], Caner Deger[3], Badri Vishal[1], Aleš Vlk[4], Pia Dally[1], Bumin K. Yildirim[1], Randi Azmi[1], Jiang Liu[1], Edward A. Jackson[5], Holly M. Johnson[2], Manting Gui[2], Henning Richter[5], Anil R. Pininti[1], Helen Bristow[1], Maxime Babics[1], Arsalan Razzaq[1], Thomas G. Allen[1], Martin Ledinský[4], Ilhan Yavuz[3], Barry P. Rand[2], Stefaan De Wolf[1,*]

[1]*King Abdullah University of Science and Technology (KAUST), KAUST Solar Center (KSC), Physical Science and Engineering Division (PSE), Thuwal 23955-6900, Kingdom of Saudi Arabia*

[2]*Department of Electrical and Computer Engineering, Princeton University, Princeton, NJ, 08544 USA*

[3]*Department of Physics, Marmara University, Istanbul, Türkiye*

[4]*Laboratory of Nanostructures and Nanomaterials, Institute of Physics, Academy of Sciences of the Czech Republic, v. v. i., Cukrovarnická 10, Prague, 162 00, Czech Republic*

[5]*Nano-C, Inc., 33 Southwest Park, Westwood, MA 02090, USA*

[⊥]These authors contributed equally to this work

*Email: ahmedali.ahmed@kaust.edu.sa, erkan.aydin@kaust.edu.sa, stefaan.dewolf@kaust.edu.sa

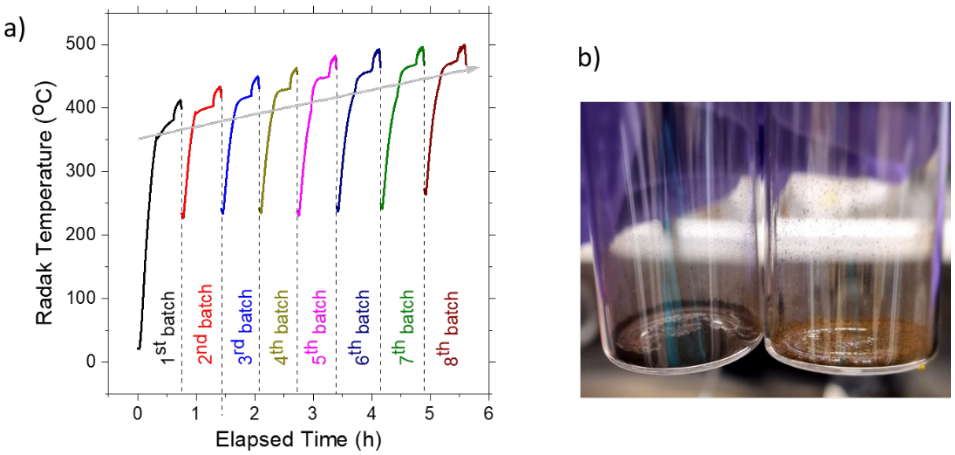

Supplementary Figure 1: a) Quartz crucible temperature profile during the deposition cycles. b) The picture of the fresh (left) and thermally cycled (right) as-received fullerene powders inside the quartz crucible. For these depositions we put 310 mg C_{60} before the 1st deposition, and we collected 50 mg as the remaining powder after the 8th deposition. We note that the deposition happens when T_C is around 350 °C (for the 1st cycle); during sample exchange T_C lowers to 240 °C, but was not cooled to room temperature, to mimic the envisaged industrial processing.

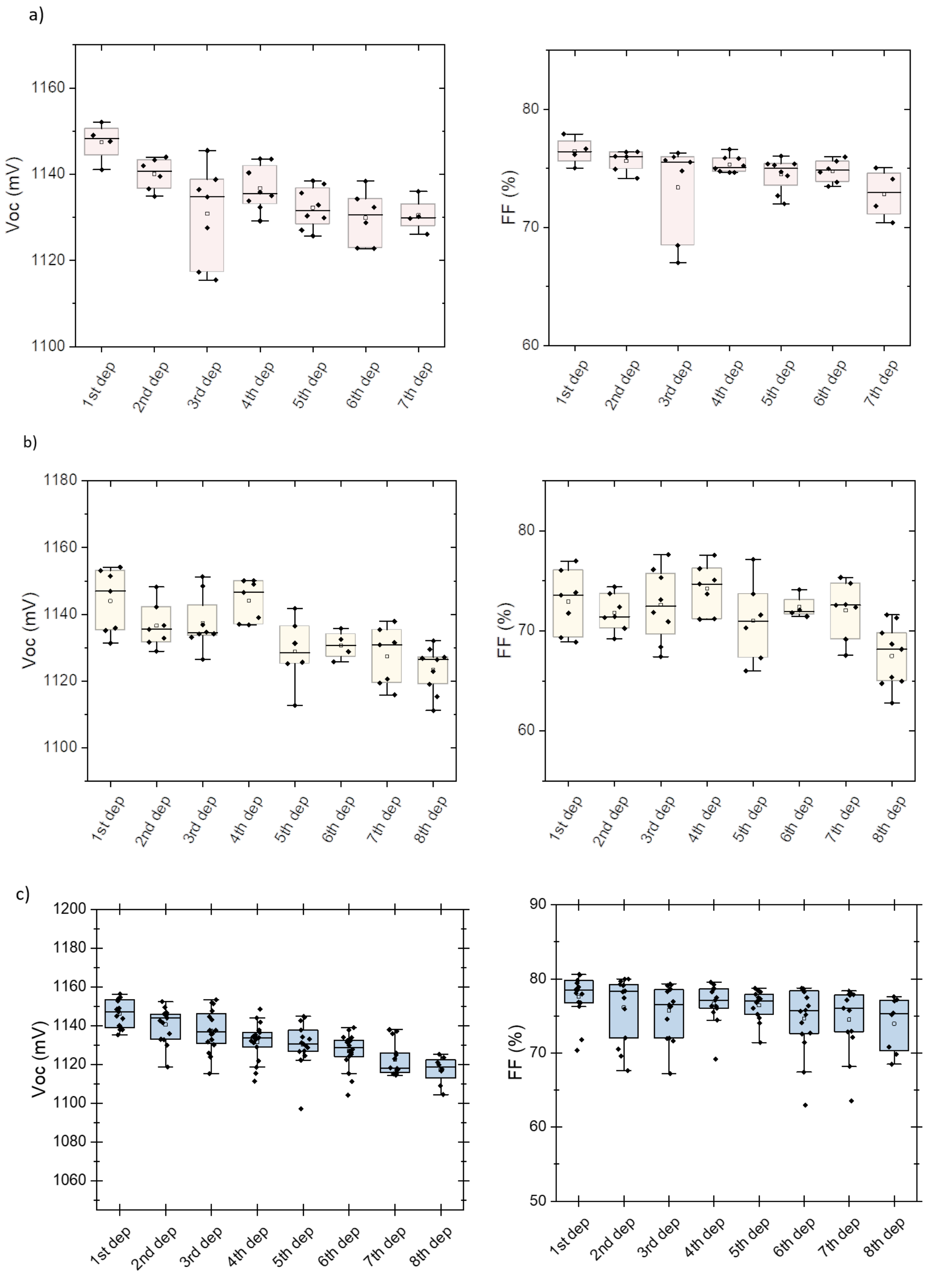

Supplementary Figure 2: Statistical distributions of V_{oc} and *FF* for single-junction perovskite solar cells based on, a) 1.68 eV bandgap one step solution-processed $Cs_{0.05}FA_{0.8}MA_{0.15}Pb(I_{0.745}Br_{0.255})_3$ perovskite absorber, b) 1.55 eV bandgap one step solution-processed $Cs_{0.03}(FA_{0.90}MA_{0.10})_{0.97}PbI_3$ perovskite absorber, and c) 1.68 eV bandgap hybrid perovskite absorber.

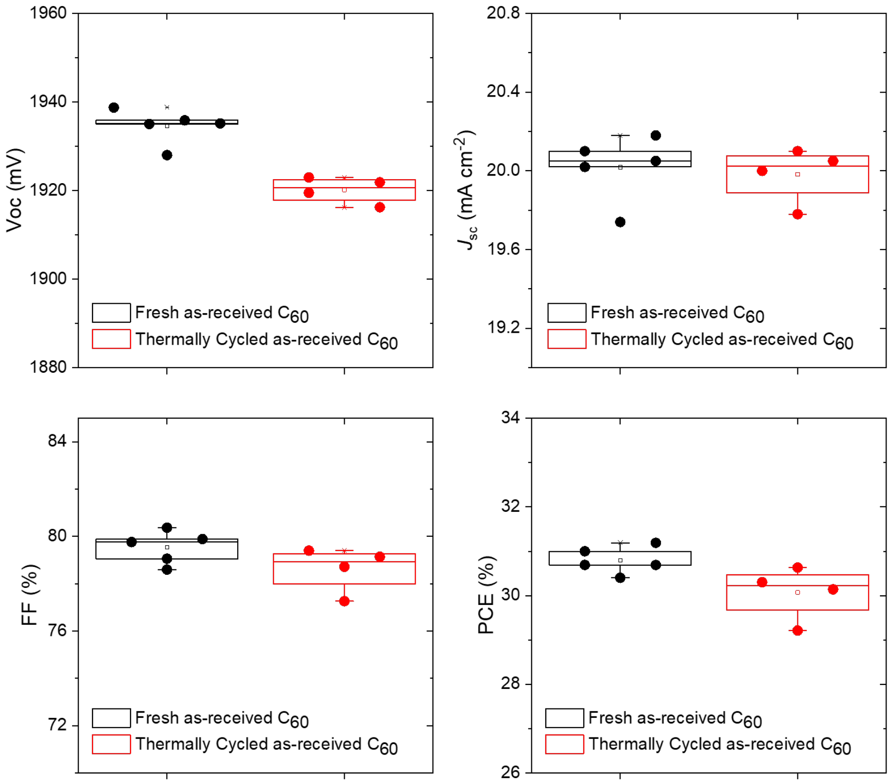

Supplementary Figure 3: Statistical distributions of the photovoltaic characteristics for perovskite/silicon tandem solar cells with fresh and thermally cycled as-received C_{60}.

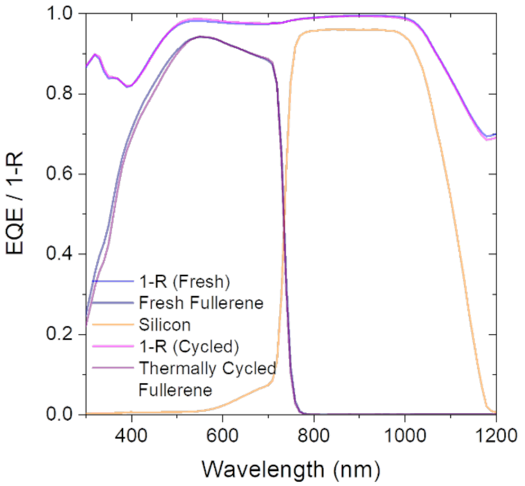

Supplementary Figure 4: EQE and 1-reflection (R) of perovskite/silicon tandem solar cells with fresh and thermally cycled as-received C_{60}.

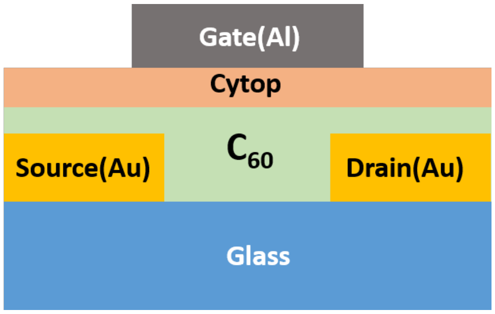

Supplementary Figure 5: Architecture of FET used to measure the electron mobility of C_{60} thin film.

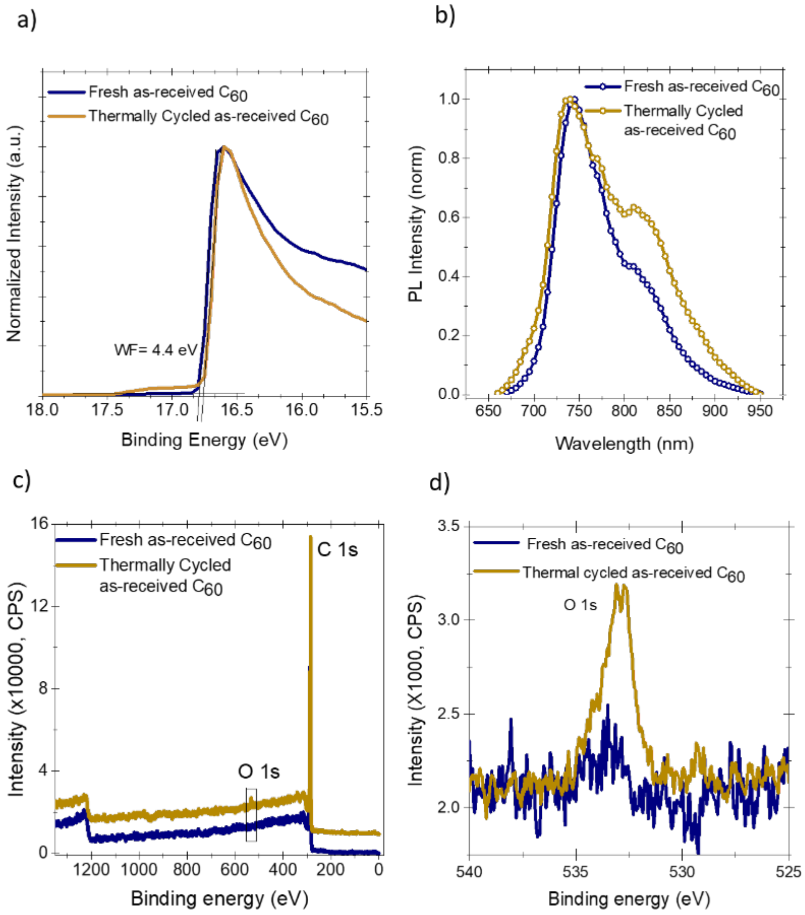

Supplementary Figure 6: a) UPS spectra of the as-received C_{60} thin films on c-Si wafer showing photoelectron cut-off region. b) PL spectra. c**)** XPS survey and d) high-resolution spectra of oxygen.

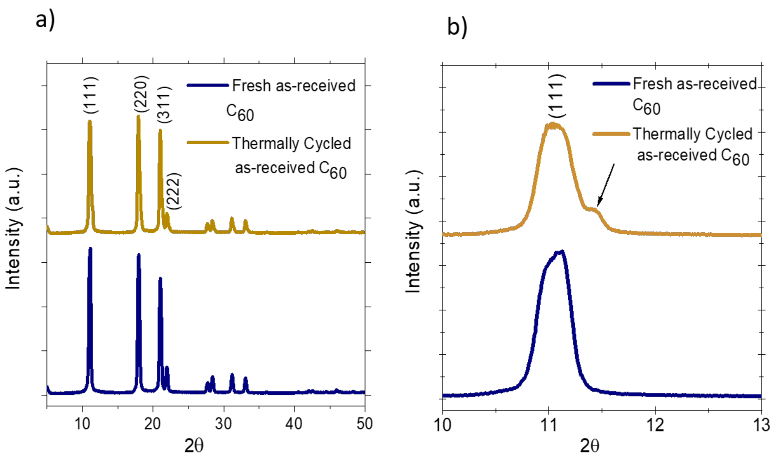

Supplementary Figure 7: a) and b) XRD of thermally cycled as-received C_{60}.

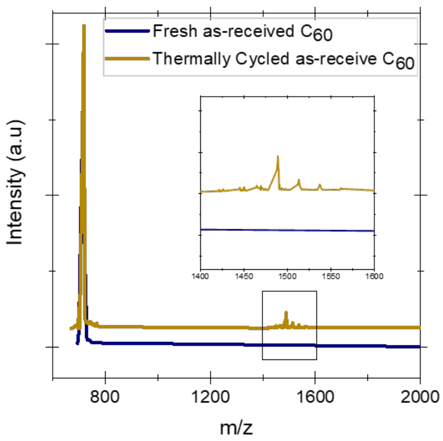

Supplementary Figure 8: MALDI-TOF analysis of fresh and thermally cycled as-received C_{60} powder.

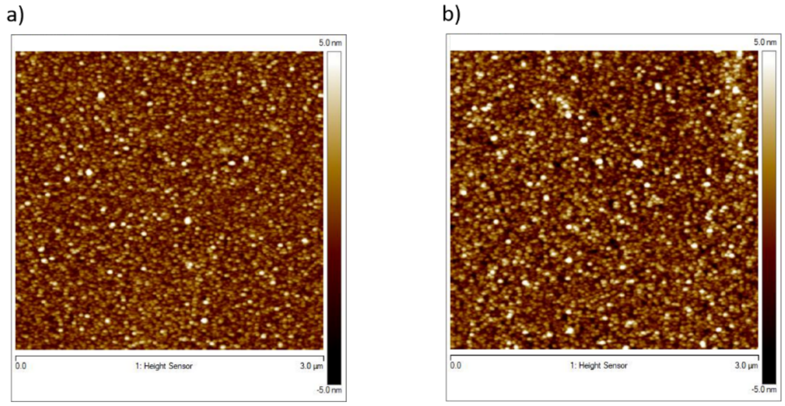

Supplementary Figure 9: AFM of a) fresh and b) thermally cycled as-received C_{60} films.

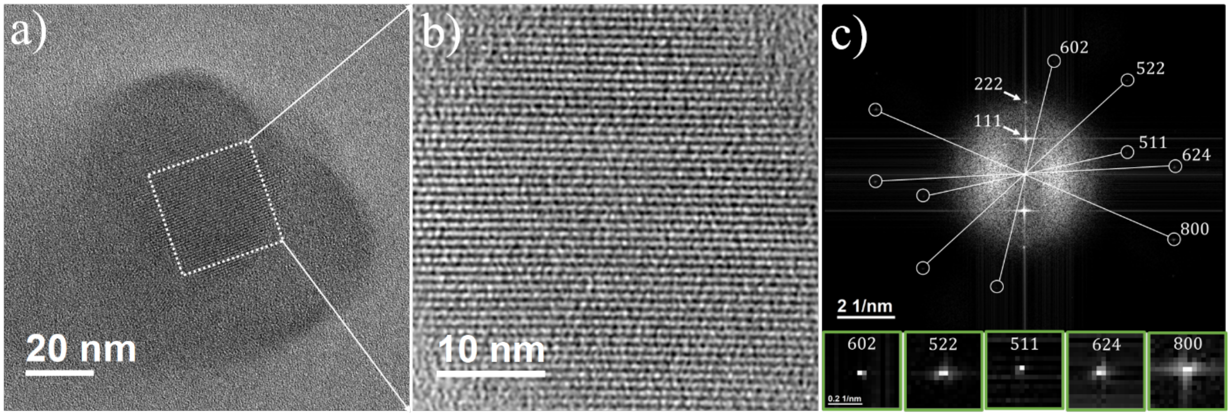

Supplementary Figure 10: a) Low magnification TEM of thermally cycled as-received C_{60}, b) High magnification filtered HRTEM, c) corresponding FFT spots matches with C_{60} d-spacing across molecularly crystalline fullerenes (insert zoom-in FFT spots).

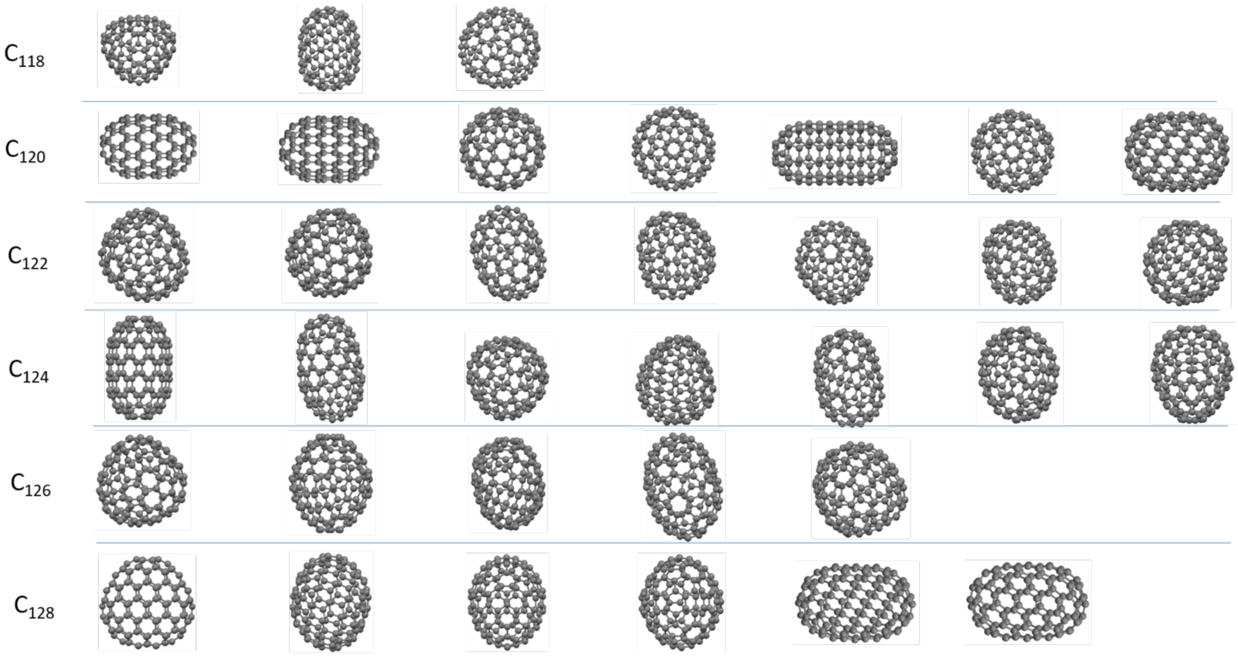

Supplementary Figure 11: Possible high symmetry configurations of fullerene derivatives for various C_n, n = 118 – 128.

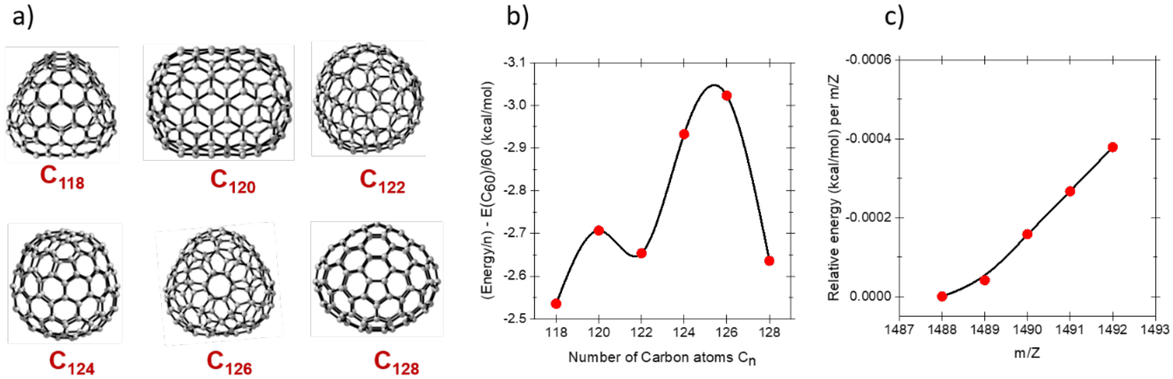

Supplementary Figure 12: a) The most stable configuration of different fullerenes via DFT calculations. b) The relation between the formation energy and different fullerenes. c) The relation between formation energy and C_{124} with different numbers of C^{13}.

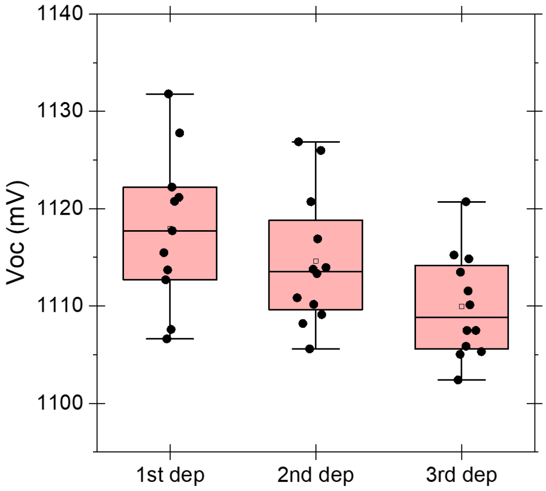

Supplementary Figure 13: Statistical distributions of V_{oc} for 1st, 2nd and 3rd depositions of fullerene with CaF_2 as interlayer between perovskite surface and as-received C_{60} layer.

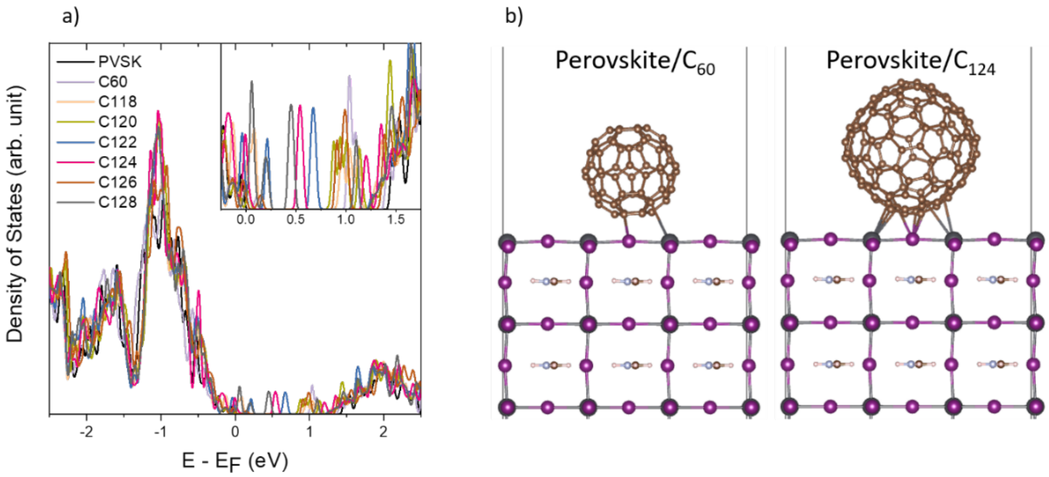

Supplementary Figure 14: a) DOS of pristine perovskite, and approaching the C_{60} and C_{118}-C_{128} towards the surface of these crystal slabs. b) Bonding between perovskite/ C_{60} and perovskite/C_{124}.

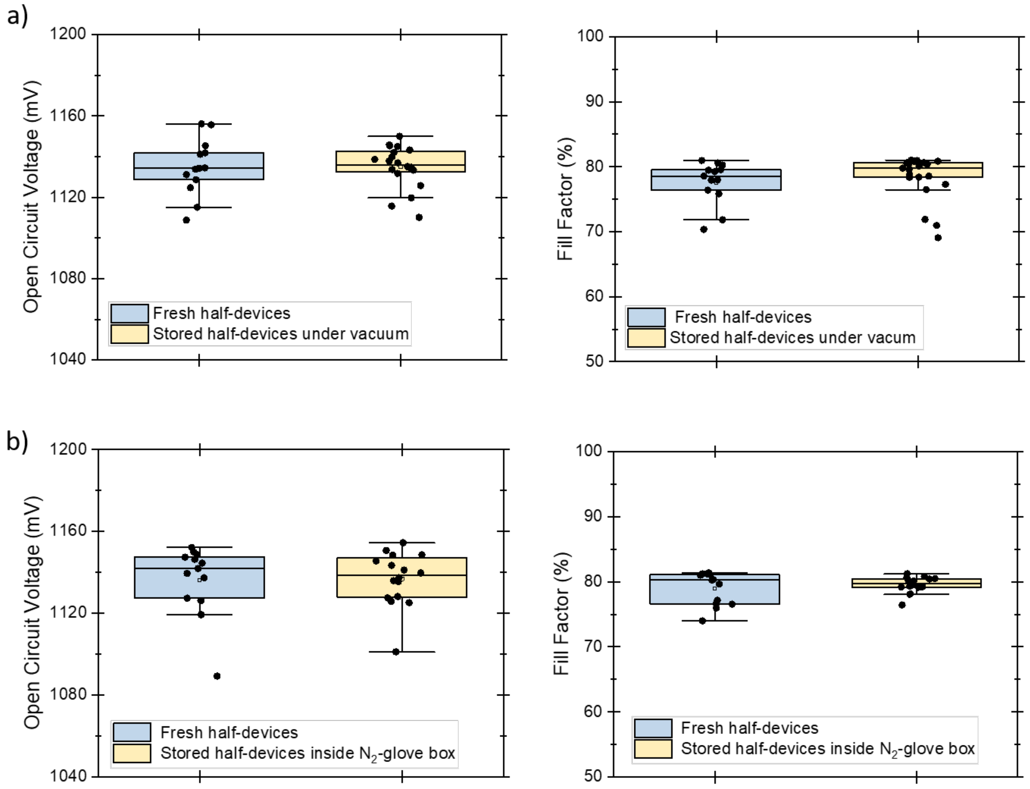

Supplementary Figure 15: Statistical distributions of V_{oc} and *FF* of fabricated solar cell devices with a) fresh half-device and stored half-devices under vacuum, 1E-3 to 1E-4 Torr , b) fresh half-devices and stored half-devices inside N_2-glovebox, O_2 <10 ppm.

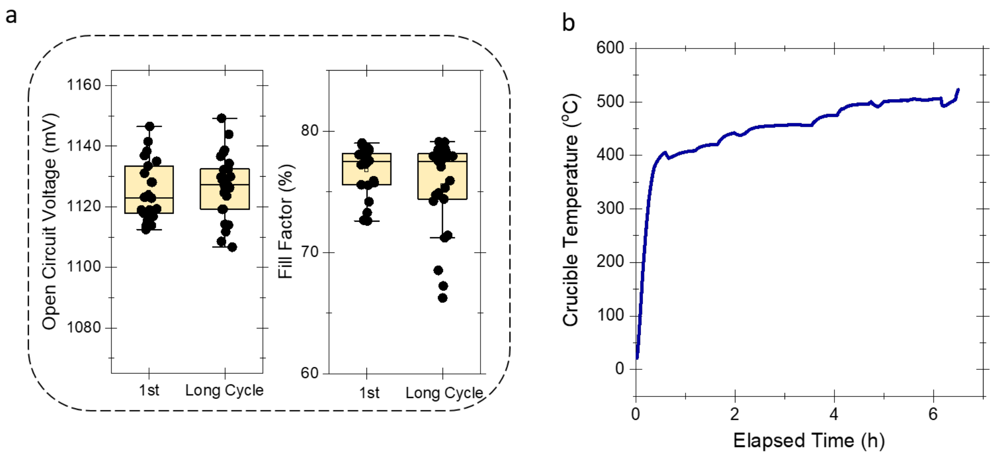

Supplementary Figure 16: a) Statistical distributions of V_{oc} and FF for the 1st and long thermal cycle. b) Quartz crucible temperature profile during the long thermal cycle.

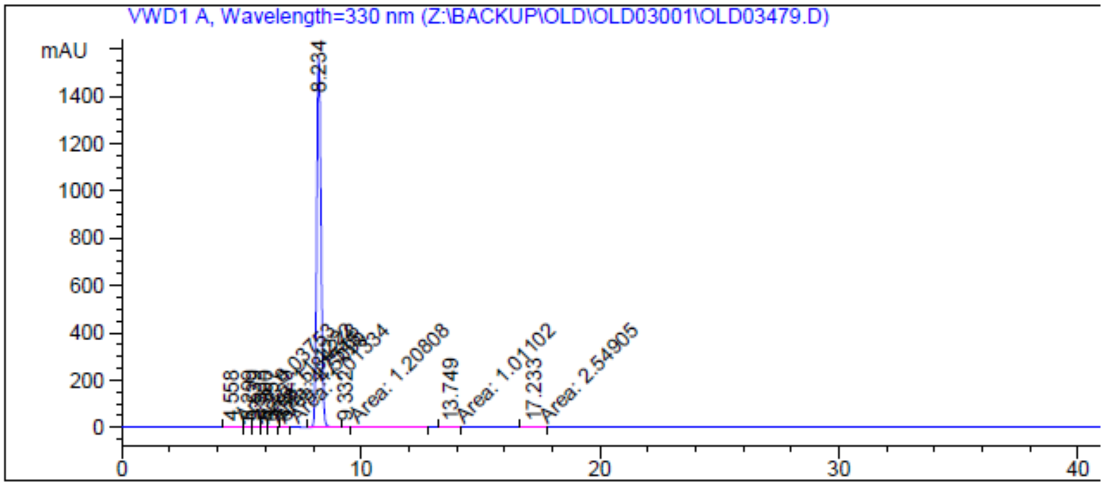


```
================================================================================
                         External Standard Report
================================================================================

Sorted By             :      Signal
Multiplier            :      1.0000
Dilution              :      1.0000
Use Multiplier & Dilution Factor with ISTDs

Signal 1: VWD1 A, Wavelength=330 nm

================================================================================
================================================================================
                           Area Percent Report
================================================================================

Sorted By             :      Signal
Multiplier            :      1.0000
Dilution              :      1.0000
Use Multiplier & Dilution Factor with ISTDs


Signal 1: VWD1 A, Wavelength=330 nm
```

Peak #	RetTime [min]	Type	Width [min]	Area mAU *s	Height [mAU]	Area %
1	4.558	MF	0.4446	8.03753	3.01319e-1	0.0407
2	5.299	FM	0.1523	11.12216	1.21740	0.0563
3	5.539	FM	0.1730	5.84278	5.62769e-1	0.0296
4	5.990	FM	0.1607	4.75450	4.92981e-1	0.0241
5	6.257	FM	0.1655	4.50990	4.54107e-1	0.0228
6	6.829	MM	0.1953	1.01334	8.64845e-2	5.126e-3
7	8.234	MM R	0.2106	1.97270e4	1561.45605	99.7974
8	9.332	MM T	0.2019	1.20808	9.97305e-2	6.112e-3
9	13.749	MM	0.3403	1.01102	4.95164e-2	5.115e-3
10	17.233	MM	0.4787	2.54905	8.87468e-2	0.0129

Supplementary Figure 17: HPLC analysis of as-received C_{60} powder.

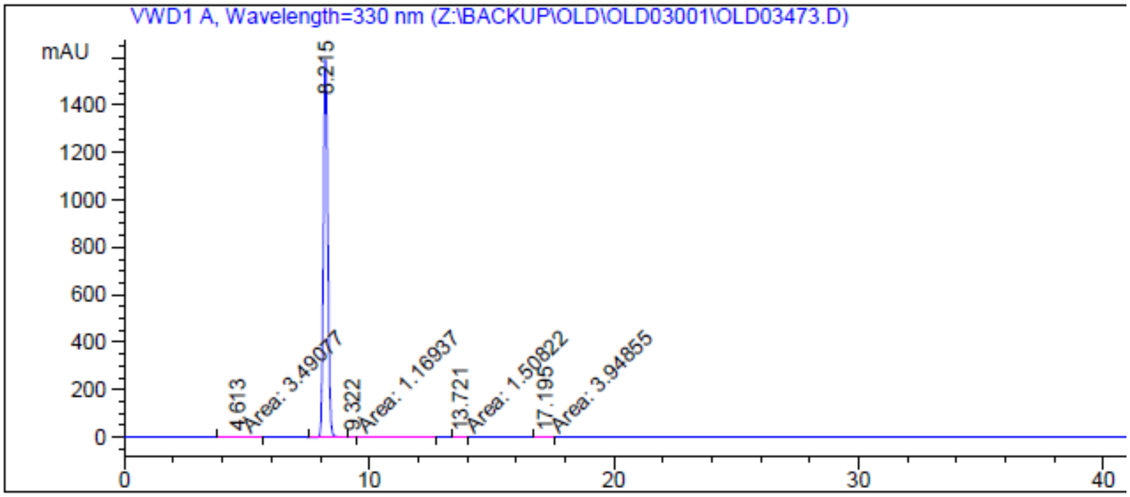


```
=====================================================================
                    External Standard Report
=====================================================================

Sorted By             :      Signal
Multiplier            :      1.0000
Dilution              :      1.0000
Use Multiplier & Dilution Factor with ISTDs

Signal 1: VWD1 A, Wavelength=330 nm

=====================================================================
=====================================================================
                    Area Percent Report
=====================================================================

Sorted By             :      Signal
Multiplier            :      1.0000
Dilution              :      1.0000
Use Multiplier & Dilution Factor with ISTDs


Signal 1: VWD1 A, Wavelength=330 nm

Peak RetTime Type  Width     Area      Height     Area
  #   [min]        [min]  mAU   *s    [mAU   ]      %
----|-------|----|-------|----------|----------|--------|
   1   4.613 MM    0.3728    3.49077 1.56057e-1   0.0176
   2   8.215 MM R  0.2079 1.98665e4  1592.26404  99.9491
   3   9.322 MM T  0.2491    1.16937 7.82481e-2 5.883e-3
   4  13.721 MM    0.3319    1.50822 7.57377e-2 7.588e-3
   5  17.195 MM    0.4574    3.94855 1.43892e-1   0.0199

Totals :                  1.98766e4  1592.71797

 Results obtained with enhanced integrator!
=====================================================================
                         *** End of Report ***
```

Supplementary Figure 18: HPLC analysis of sublimed C_{60} powder.

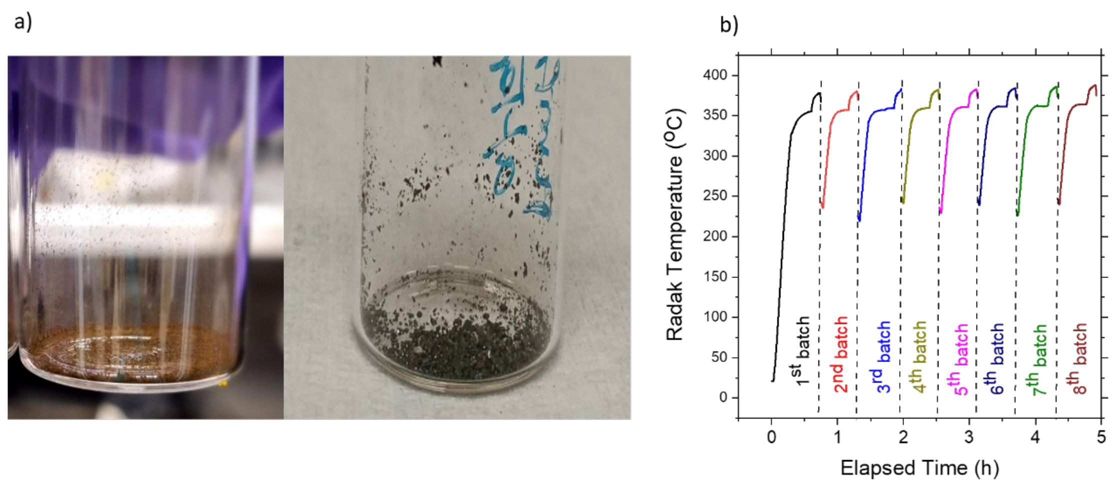

Supplementary Figure 19: a) The picture of the thermally cycled as-received C_{60} (left) and thermally cycled sublimed C_{60} (right). b) Quartz crucible temperature profile during the deposition cycles of sublimed C_{60}. For these depositions, we put 310 mg C_{60} powder inside the crucible.

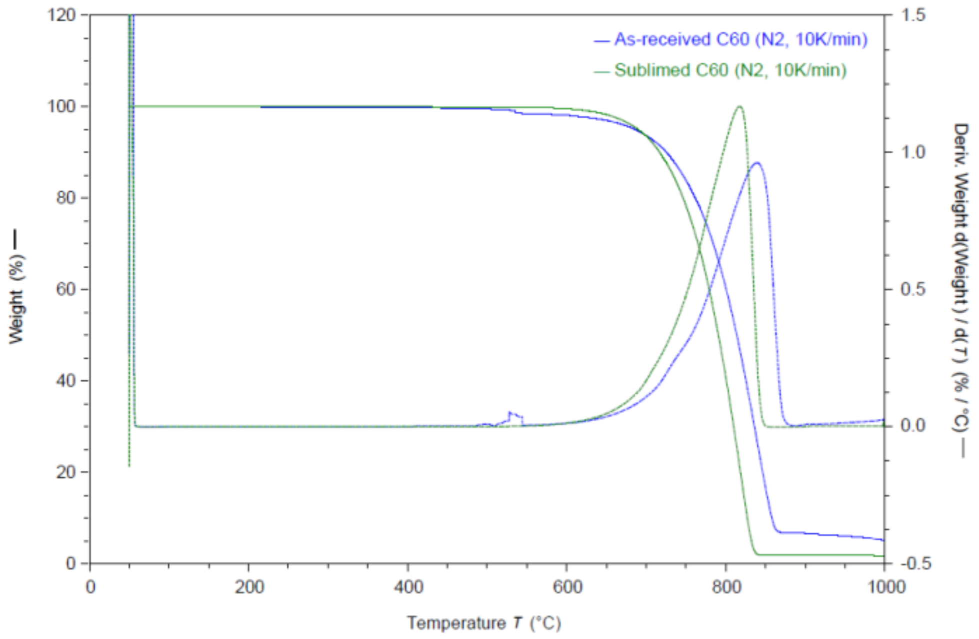

Supplementary Figure 20: TGA analysis of as-received C_{60} (blue) and sublimed C_{60} (green).

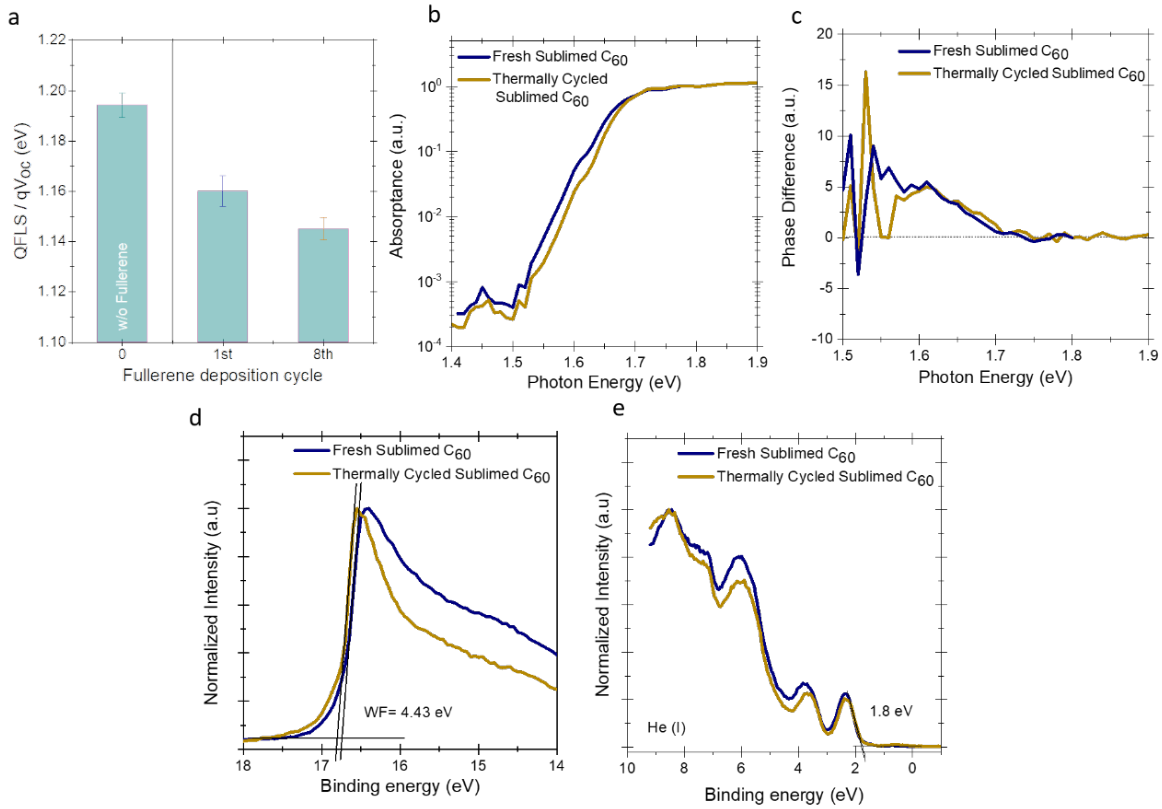

Supplementary Figure 21. a) QFLS values for the stack of ITO/NiO_x/MeO-2PACz/perovskite/ sublimed C_{60} at various sublimed C_{60} deposition cycles, together with without C_{60} stack. b) PDS absorption spectra of the quartz/perovskite/ sublimed C_{60} stacks, and c) its phase shift compared to excitation. UPS spectra of the sublimed C_{60} thin films on c-Si wafer showing the, d) photoelectron cut-off region and, e) the distribution of the energy states close to the HOMO level.

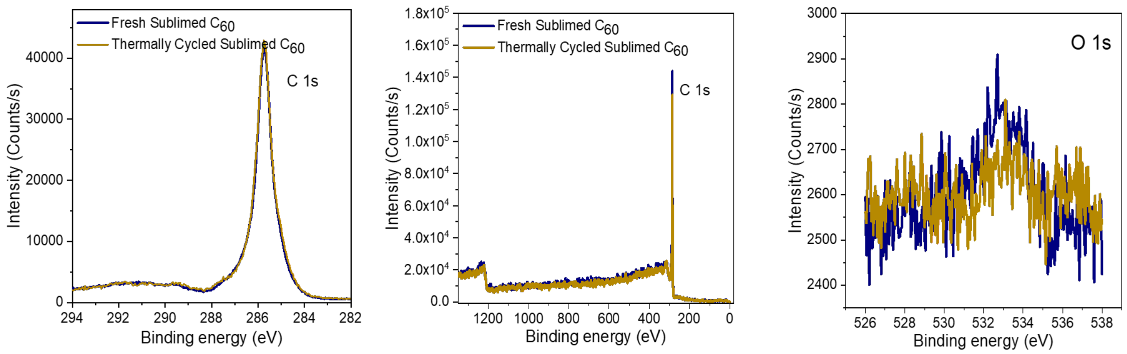

Supplementary Figure 22: XPS survey of fresh and thermally cycled sublimed C_{60}.

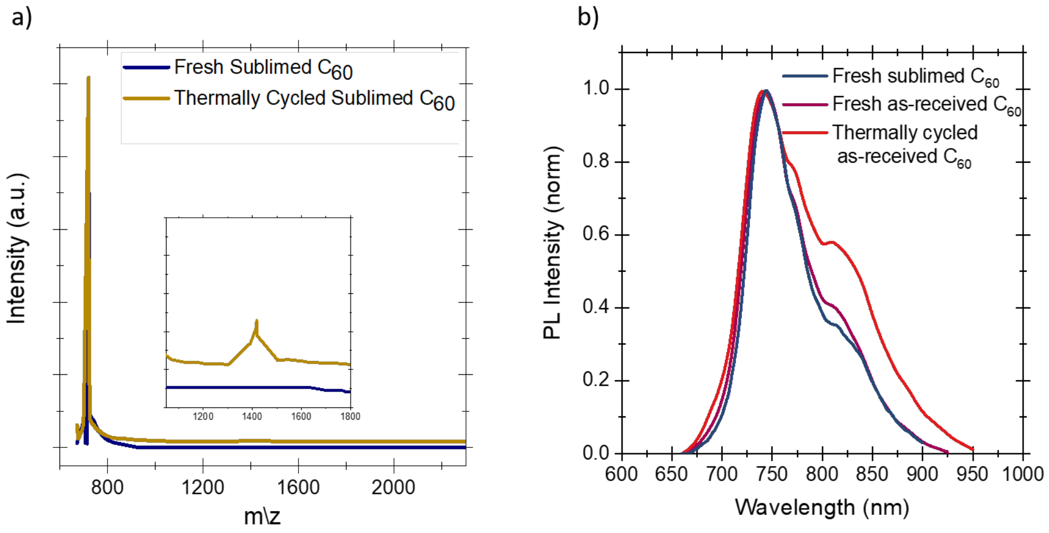

Supplementary Figure 23: a) MALDI-TOF analysis, results for fresh and thermally cycled sublimed C_{60}, b) PL of fresh and thermally cycled as-received and sublimed C_{60}.

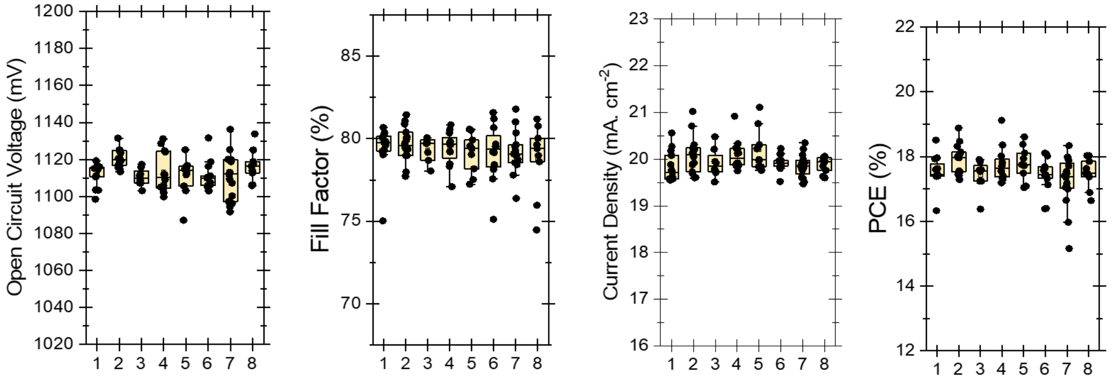

Supplementary Figure 24: Statistical distributions of the photovoltaic characteristics for single junction PSCs with repeated deposition cycles of sublimed C_{60}. Slightly lower V_{oc} is due to the batch-to-batch variation.

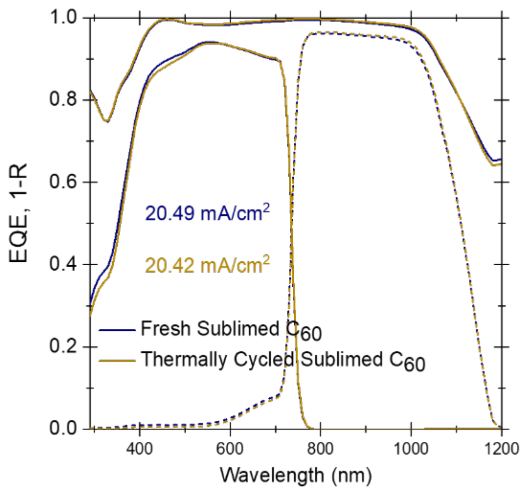

Supplementary Figure 25: EQE and 1-R of perovskite/silicon tandem solar cells using fresh and thermally cycled sublimed C_{60}.

Fraunhofer ISE CalLab PV Cells

Heidenhofstr.2

79110 Freiburg

Werkskalibrierschein
Proprietary calibration report

10001019KAU0223

Gegenstand<br>*Object*	**monofacial tandem solar cell**
Hersteller<br>*Manufacturer*	**KAUST**
Typ<br>*Type*	**PSC/Si**
Fabrikat/Serien-Nr.<br>*Serial number*	**KAU001 / DT194-04-07**
Auftraggeber<br>*Customer*	**King Abdullah University of Science & Technology**<br>**Discovery Boulevard, Al Kindi Building, Level 3**<br>**23955 Jeddah**<br>**Kingdom of Saudi Arabia**
Auftragsnummer<br>*Order No.*	**019KAU0223**
Anzahl der Seiten<br>*Number of pages*	**6**
Datum der Kalibrierung<br>*Date of calibration*	**16.02.2023**

Kalibrierscheine ohne Unterschrift haben keine Gültigkeit. *Calibration certificates without signature are not valid.*

Datum<br>*Date*	Leiter des Kalibrierlaboratoriums<br>*Head of the calibration laboratory*	Bearbeiter<br>*Person in charge*
21.02.2023	*Jochen Hohl-Ebinger*	*Astrid Semeraro*

Fraunhofer ISE

CalLab PV Cells

10001019KAU0223

Die Rückführung der Spektralmessung auf SI-Einheiten erfolgte über den Vergleich mit einer Standardlampe.
The traceability of the measurement of the spectral distribution to SI-Units is achieved using a standard lamp for the calibration of the spectroradiometer.

Identitäts-Nr. / *Identity-Nr.* :	Kalibrierschein-Nr./ *Certificate-Nr.* :	Rückführung/ *Traceability* :
BN-9101-451	40006-20-PTB	PTB

3. Messbedingungen

Measurement conditions

Standardtestbedingungen (STC) / *Standard Testing Conditions (STC)* :

Absolute Bestrahlungsstärke / *Total irradiance* :	1000	W/m²
Nominalwert der Temperatur des Messobjektes / *Nominal Value of Temperature of the DUT* :	25	°C
Spektrale Bestrahlungsstärke / *Spectral irradiance distribution* :	AM1.5G Ed.4 (2019)	

Die Messung der IV-Kennlinie (Strom-Spannungs-Kennlinie) des Messobjektes erfolgt mit Hilfe eines Vierquadranten-Netzteiles und eines Kalibrierwiderstandes. Die Temperatur der Solarzelle wird mit einem Tastsensor ermittelt und auf (25±0,5)°C eingestellt.
The measurement of the IV-curve is performed with a 4-quadrant power amplifier and a calibration resistor. The temperature of the solar cell is determined by a sensor and adjusted to (25±0.5)°C.

4. Messergebnis

Measurement results

Fläche / *Area* (da)[1]: = (1.0005 ± 0.0062) cm²

[1] : *(t) = total area, (ap) = aperture area, (da) = designated illumination area /6/*

Kennlinienparameter des Messobjektes unter Standardtestbedingungen (STC) / *IV-curve parameter under Standard Testing Conditions (STC)* :

		Vorwärtsrichtung / *forwards scan direction*	Rückwärtsrichtung / *reverse scan direction*	steady state MPP
V_{oc}	=	(1962 ± 20) mV	(1960 ± 20) mV	
I_{SC} (Ed.2 - 2008)	=	(20.40 ± 0.39) mA	(20.44 ± 0.39) mA	
I_{MPP}	=	17.99 mA	18.43 mA	(18.37 ± 0.63) mA
V_{MPP}	=	1687 mV	1665 mV	(1682 ± 35) mV
P_{MPP}	=	30.3 mW	30.7 mW	(30.9 ± 1.3) mW
FF	=	75.8 %	76.6 %	
η	=			(30.9 ± 1.3) %

Fraunhofer ISE

CalLab PV Cells

10001019KAU0223

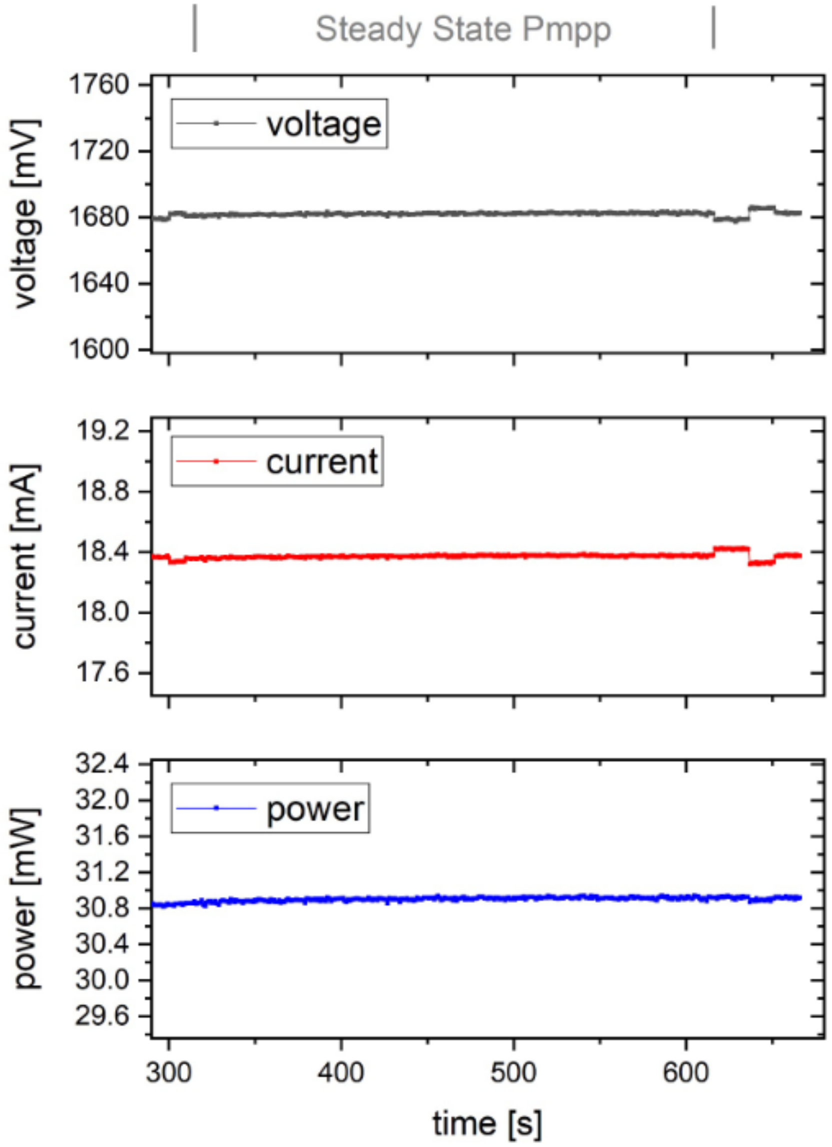

Supplementary Figure 26: Certification report by Fraunhofer Institute of Solar Energy (ISE). The area is (1.0005 ± 0.0062) cm^2. Date of calibration: 21.02.2023
