# Supplementary material for: Sublimed C60 for efficient and repeatable perovskite-based solar cells
Source: Nat Commun. 2024 Jan 24;15:708. doi: 10.1038/s41467-024-44974-0 (PMC10808237; doi:10.1038/s41467-024-44974-0)
Supplement: Supplementary file 3 — Reporting Summary [file 41467_2024_44974_MOESM3_ESM.pdf]

Corresponding author(s): Ahmed Ali Said
Erkan Aydin
Stefaan De Wolf

Solar Cells Reporting Summary

Nature Research wishes to improve the reproducibility of the work that we publish. This form is intended for publication with all accepted papers reporting the characterization of photovoltaic devices and provides structure for consistency and transparency in reporting. Some list items might not apply to an individual manuscript, but all fields must be completed for clarity.

For further information on Nature Research policies, including our data availability policy, see Authors & Referees.

Experimental design

Please check: are the following details reported in the manuscript?

1. Dimensions

Item	Yes/No	Details
Area of the tested solar cells	☒ Yes ☐ No	For single junction cell, the area (0.1 cm2)<br>For tandem certified area (1.0005 cm2), see supplementary Figure S23. For tandem in-house measurement (1.04 cm2).<br>*Explain why this information is not reported/not relevant.*
Method used to determine the device area	☒ Yes ☐ No	For in-house measurement, shadow mask is used to determine the active area<br>Certified tandem is measured independently by Fraunhofer ISE CalLab<br>*Explain why this information is not reported/not relevant.*

2. Current-voltage characterization

Item	Yes/No	Details
Current density-voltage (J-V) plots in both forward and backward direction	☒ Yes ☐ No	Given in Figure 1b and e. Figure 4c and f<br>*Explain why this information is not reported/not relevant.*
Voltage scan conditions<br>*For instance: scan direction, speed, dwell times*	☒ Yes ☐ No	Given in solar cell characterization section in Methods<br>*Explain why this information is not reported/not relevant.*
Test environment<br>*For instance: characterization temperature, in air or in glove box*	☒ Yes ☐ No	Given in solar cell characterization section in Methods<br>*Explain why this information is not reported/not relevant.*
Protocol for preconditioning of the device before its characterization	☐ Yes ☒ No	*State where this information can be found in the text.*<br>*Explain why this information is not reported/not relevant.*
Stability of the J-V characteristic<br>*Verified with time evolution of the maximum power point or with the photocurrent at maximum power point; see ref. 7 for details.*	☒ Yes ☐ No	J-V measurements were performed after the cell had undergone maximum powerpoint tracking for 200-300 s. This was done to ensure that the cell reached a stable steady-state efficiency during J-V characterization.<br>*Explain why this information is not reported/not relevant.*

3. Hysteresis or any other unusual behaviour

Item	Yes/No	Details
Description of the unusual behaviour observed during the characterization	☐ Yes ☒ No	*State where this information can be found in the text.*<br>*Explain why this information is not reported/not relevant.*
Related experimental data	☒ Yes ☐ No	The negligible hysteresis was observed in J-V plots for the reverse and forwards scans.<br>*Explain why this information is not reported/not relevant.*

4. Efficiency

Item	Yes/No	Details
External quantum efficiency (EQE) or incident photons to current efficiency (IPCE)	☒ Yes ☐ No	Given in Supplementary Figure 4 and 25<br>*Explain why this information is not reported/not relevant.*
A comparison between the integrated response under the standard reference spectrum and the response measure under the simulator	☐ Yes ☒ No	*State where this information can be found in the text.*<br>*Explain why this information is not reported/not relevant.*
For tandem solar cells, the bias illumination and bias voltage used for each subcell	☒ Yes ☐ No	Relevant information is provided in Methods.<br>*Explain why this information is not reported/not relevant.*

5. Calibration

Light source and reference cell or sensor used for the characterization

☒ Yes ☐ No

AAA class LED-based solar simulator was used for in-house measurements, and the intensity is corrected by reference cell calibrated by Fraunhofer ISE.

Explain why this information is not reported/not relevant.

Confirmation that the reference cell was calibrated and certified

☒ Yes ☐ No

Farunhofer ISE003/020075 CalLab certified c-Si cell.

Explain why this information is not reported/not relevant.

Calculation of spectral mismatch between the reference cell and the devices under test

☐ Yes ☒ No

State where this information can be found in the text.

Explain why this information is not reported/not relevant.

6. Mask/aperture

Size of the mask/aperture used during testing

☒ Yes ☐ No

Given in solar cell characterization section Methods.

Explain why this information is not reported/not relevant.

Variation of the measured short-circuit current density with the mask/aperture area

☐ Yes ☒ No

State where this information can be found in the text.

We report our results always with aperture. Edge effects are minimized. Fraunhofer ISE CalLab measured the aperture area by themselves (Supplementary Figure 26.)

7. Performance certification

Identity of the independent certification laboratory that confirmed the photovoltaic performance

☒ Yes ☐ No

Fraunhofer ISE CalLab

Explain why this information is not reported/not relevant.

A copy of any certificate(s)
Provide in Supplementary Information

☒ Yes ☐ No

Given in Supplementary Figure 26

Explain why this information is not reported/not relevant.

8. Statistics

Number of solar cells tested

☒ Yes ☐ No

For different conditions, different number of devices were fabricated. For single junction cell > 6 device fabricated for each condition to show the reproducibility. For Tandem cell 4 device fabricated for each condition to show the reproducibility.

Explain why this information is not reported/not relevant.

Statistical analysis of the device performance

☒ Yes ☐ No

Given in Figure 1C, Figure 4a,b,d and e.
Supplementary Figure 2,3,13, 15,16 and 24

Explain why this information is not reported/not relevant.

9. Long-term stability analysis

Type of analysis, bias conditions and environmental conditions
For instance: illumination type, temperature, atmosphere humidity, encapsulation method, preconditioning temperature

☐ Yes ☒ No

State where this information can be found in the text.

The focus of this study is not stability.
